# Supplementary material for: Replication dynamics identifies the folding principles of the inactive X chromosome
Source: Nat Struct Mol Biol. 2023 Aug 10;30(8):1224–37. doi: 10.1038/s41594-023-01052-1 (PMC10442229; doi:10.1038/s41594-023-01052-1)

nature structural & molecular biology

Article

https://doi.org/10.1038/s41594-023-01052-1

Replication dynamics identifies the folding principles of the inactive X chromosome

In the format provided by the authors and unedited

Table of Contents

Supplementary Figures 2

Supplementary Figure 1 2

Supplementary Figure 2 5

Supplementary Figure 3 7

Supplementary Figure 4 9

Supplementary Figure 5 11

Supplementary Figure 6 14

Supplementary Figure 7 16

Supplementary Figure 8 18

Supplementary Figure 9 20

Supplementary Figure 10 22

Supplementary Texts 24

Supplementary Text 1 24

Supplementary Text 2 25

Supplementary Text 3 26

Description of Supplementary Tables 27

Supplementary References 28

Supplementary Data 30

Supplementary Data 1 30

Supplementary Data 2 31

Supplementary Data 3 32

Supplementary Figure 1

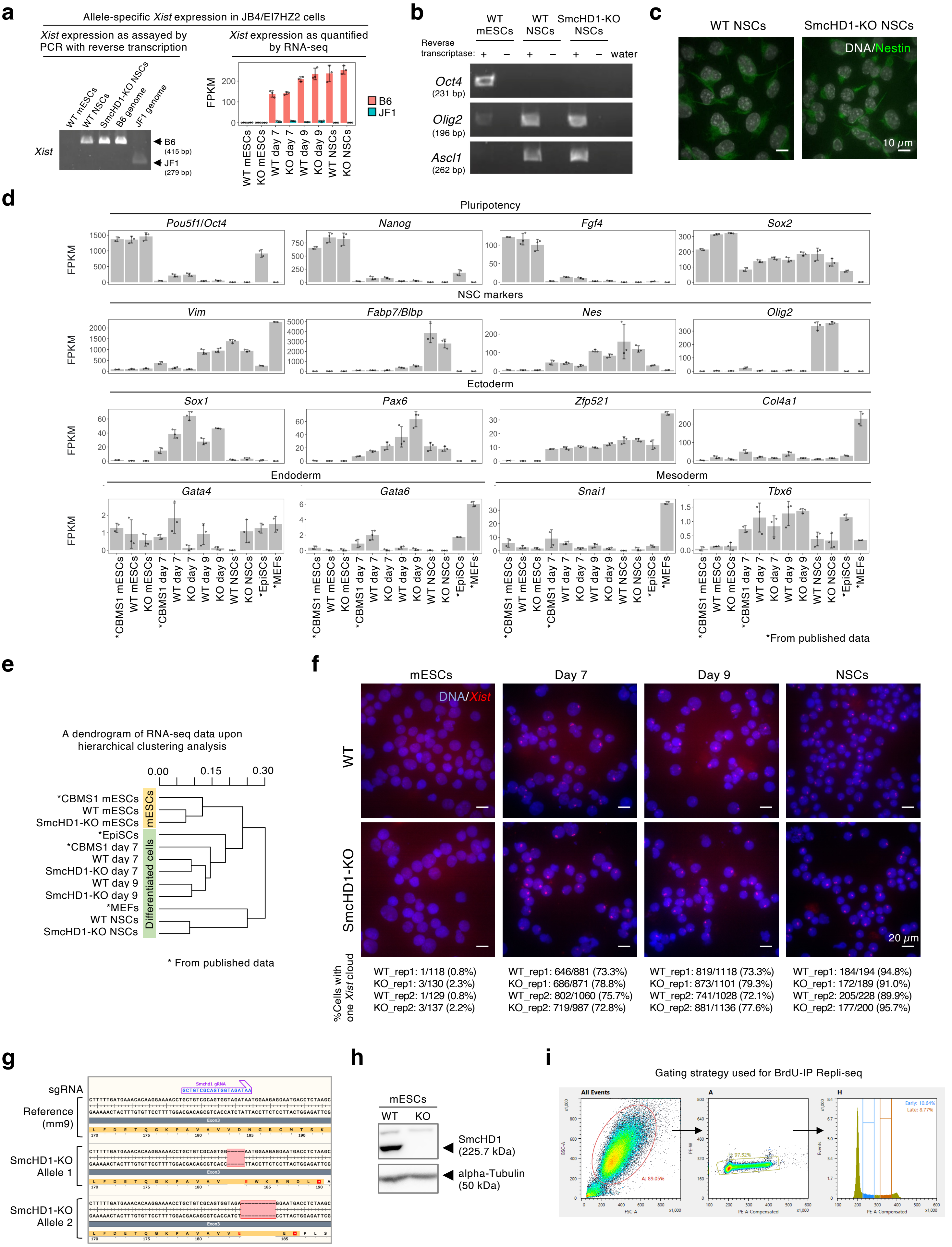

Supplementary Figure 1. Validation of cell lines used in the study

(**a**) Allele-specific *Xist* expression in day 7 or 9 differentiated cells and NSCs as assayed by PCR with reverse transcription and RNA-seq. *Xist* is expressed from the B6-X (Xi) but not from the JF1-X (Xa) in differentiated cells, confirming skewed XCI upon differentiation of mESCs. One replicate was performed for PCR with reverse transcription. Bar plots show average FPKM values of *Xist* with error bars indicating standard deviation, and each dot represents biological replicate data obtained from three replicates. (**b**) Comparison of mESCs and NSCs by PCR with reverse transcription. JB4/EI7HZ2 mESCs expressed pluripotency markers *Pou5f1* (*Oct4*), but not NSC markers *Oligo2* and *Ascl1*. In contrast, NSCs derived from these mESC clones expressed NSC markers but not pluripotency markers. (+) and (−) indicate the presence and absence of reverse transcriptase in the reaction, respectively. One replicate was performed. (**c**) Immunostaining confirmed expression of the NSC marker Nestin in wild-type (WT) and SmcHD1-mutant (KO) NSCs. Two biologically independent replicates were performed and gave similar results. (**d**) Expression profiles of markers of pluripotency, NSCs, ectoderm, endoderm, and mesoderm in differentiation intermediates derived from JB4/EI7HZ2 mESCs (this study), CBMS1 mESCs, CBMS1 day 7, embryo-derived EpiSCs, and MEFs, as assayed by RNA-seq. Bar plots show average FPKM values of genes with error bars indicating standard deviation, and each dot represents biological replicate data. Three biologically independent experiments were done for JB4/EI7HZ2 mESCs and their differentiation intermediates. Two biologically independent sets of CBMS1, EpiSCs and MEFs data are from Miura et al[1]. (**e**) Comparison of RNA-seq profiles of differentiation intermediates derived from JB4/EI7HZ2 mESCs (this study), CBMS1 mESCs, CBMS1 day 7, embryo-derived EpiSCs, and MEFs by hierarchical clustering. Average FPKM values over all genes excluding X-linked genes from two or three replicates were used to plot a dendrogram of Jensen-Shannon distances between samples. Undifferentiated mESCs and differentiated cells were clearly separated. RNA-seq profiles of cell-type-matched WT and SmcHD1-mutant (KO) cells were similar. Day 7 or 9 differentiated JB4/EI7HZ2 resembled CBMS1 day 7 and EpiSCs, which were separated from NSCs and MEFs. CBMS1, EpiSCs and MEFs data are from Miura et al[1]. (**f**) Uniform differentiation and XCI state among cells revealed by *Xist* RNA-FISH. *Xist* clouds were absent in the undifferentiated JB4/EI7HZ2 mESC population. Approximately ~75% of WT and SmcHD1-mutant (KO) JB4/EI7HZ2 cells exhibited *Xist* clouds after differentiation for 7-9 days, suggesting nearly uniform differentiation and XCI state among cells. Two biologically independent replicates were performed and gave similar results. (**g**) A single guide RNA targeting the ATPase domain (exon 3) of *SmcHD1*. Sanger

sequencing confirming the presence of biallelic nonsense mutations. Microdeletions were highlighted in red. (**h**) Western blot confirming the absence of SmcHD1 protein in SmcHD1-mutant (KO) mESCs. Two biologically independent replicates were performed and gave similar results. (**i**) BrdU-IP Repli-seq gating strategy. FSC and BSC were used for gating viable cells. PE (PI) fluorescence was then used to exclude doublet cells. The gates for sorting the early- and late-S phase cells were defined on the PE (PI) histogram.

Supplementary Figure 2

a

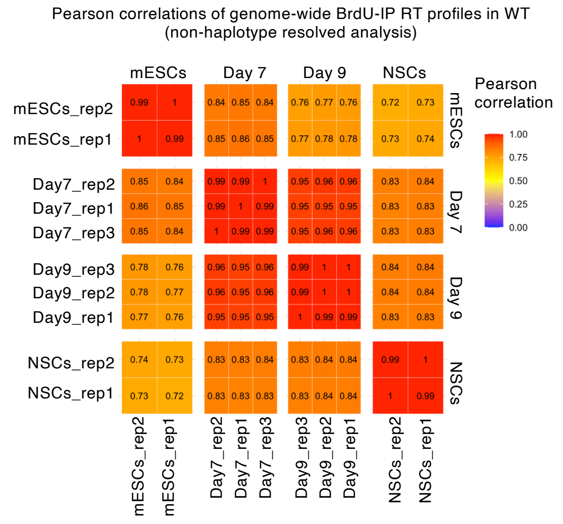

b

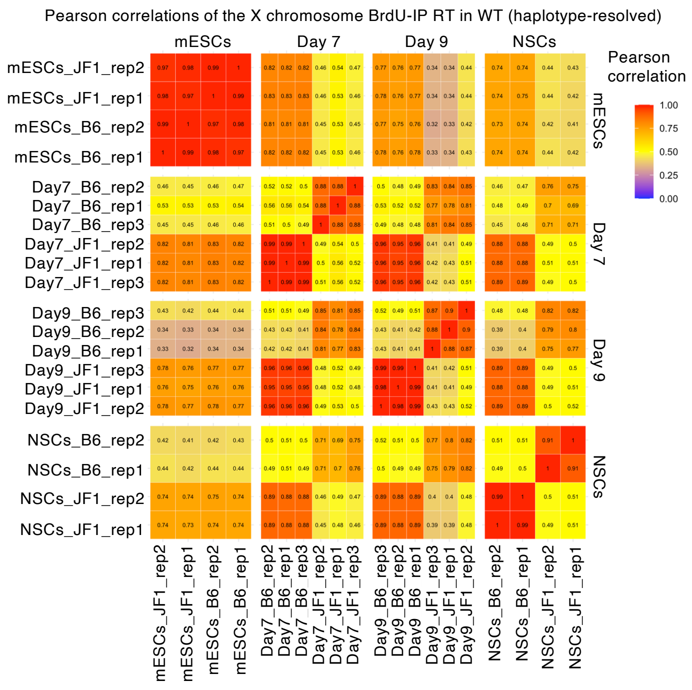

c

RT classification during mESC-to-NSC differentiation

B6-X	RT class	Bins	Mb	JF1-X	RT class	Bins	Mb
mESCs	Early	102	40.8	mESCs	Early	101	40.4
	Late	229	91.6		Late	230	92.0
	Total	331	132.4		Total	331	132.4
NSCs	Early	3	1.2	NSCs	Early	93	37.2
	Late	328	131.2		Late	238	95.2
	Total	331	132.4		Total	331	132.4
ESC-to-NSC RT changes	EtoL	99	39.6	ESC-to-NSC RT changes	EtoL	29	11.6
	LtoE	0	0		LtoE	69	27.6
	EtoE	3	1.2		EtoE	20	12.0
	LtoL	229	91.6		LtoL	206	82.4
	n/a	0	0		n/a	7	2.8
	Total	331	132.4		Total	331	132.4

d

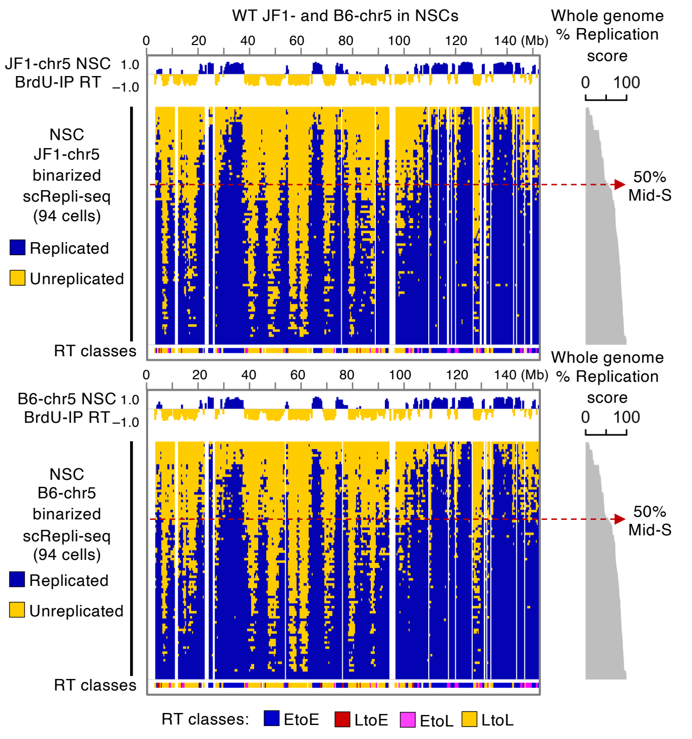

e

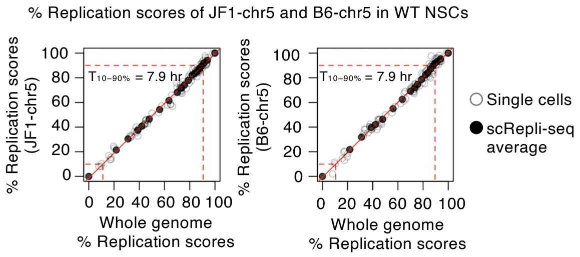

f

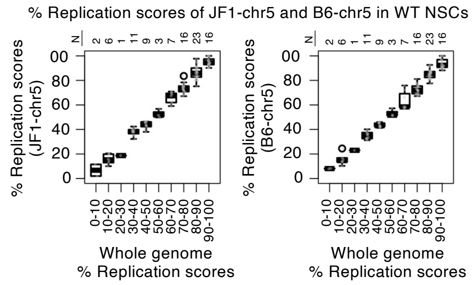

Supplementary Figure 2. BrdU-IP and scRepli-seq RT profiles of WT cells during mESC differentiation

(**a**) Pearson correlations for pair-wise comparisons of non-haplotype-resolved genome-wide BrdU-IP RT profiles (sliding windows of 200 kb at 80-kb intervals, excluding the X) of differentiation intermediates derived from JB4/EI7HZ2 mESCs. (**b**) Pearson correlations for pair-wise comparisons of haplotype-resolved JF1-X and B6-X BrdU-IP RT profiles (400-kb bins) of differentiation intermediates derived from JB4/EI7HZ2 mESCs, which revealed clear differences between the JF1-X and the B6-X in differentiated cells but not mESCs. (**c**) Classification of developmental RT regulation during mESC-to-NSC differentiation. Allele-specific RT profiles at 400-kb resolution were used for the analysis. (**d**) Binarized whole-S scRepli-seq profiles of the JF1-chr5 and the B6-chr5 from 94 WT NSCs throughout the S-phase. The scRepli-seq profiles are ordered by their percentage replication scores of the whole genome (non-haplotype-resolved, excluding the X chromosomes). BrdU-IP RT data and RT classes are shown for comparison. (**e**) Comparison of percentage replication scores of the JF1-chr5 and the B6-chr5 with those of the whole genome as in Figure 1f. $T_{10\text{-}90\%}$ values of the chromosome were obtained by linear fitting (assuming a 10 h S-phase). (**f**) Cells were divided into ten groups by their percentage replication scores and the boxplots show distributions of percentage replication scores of the JF1-chr5 and the B6-chr5 in each group.

Supplementary Figure 3

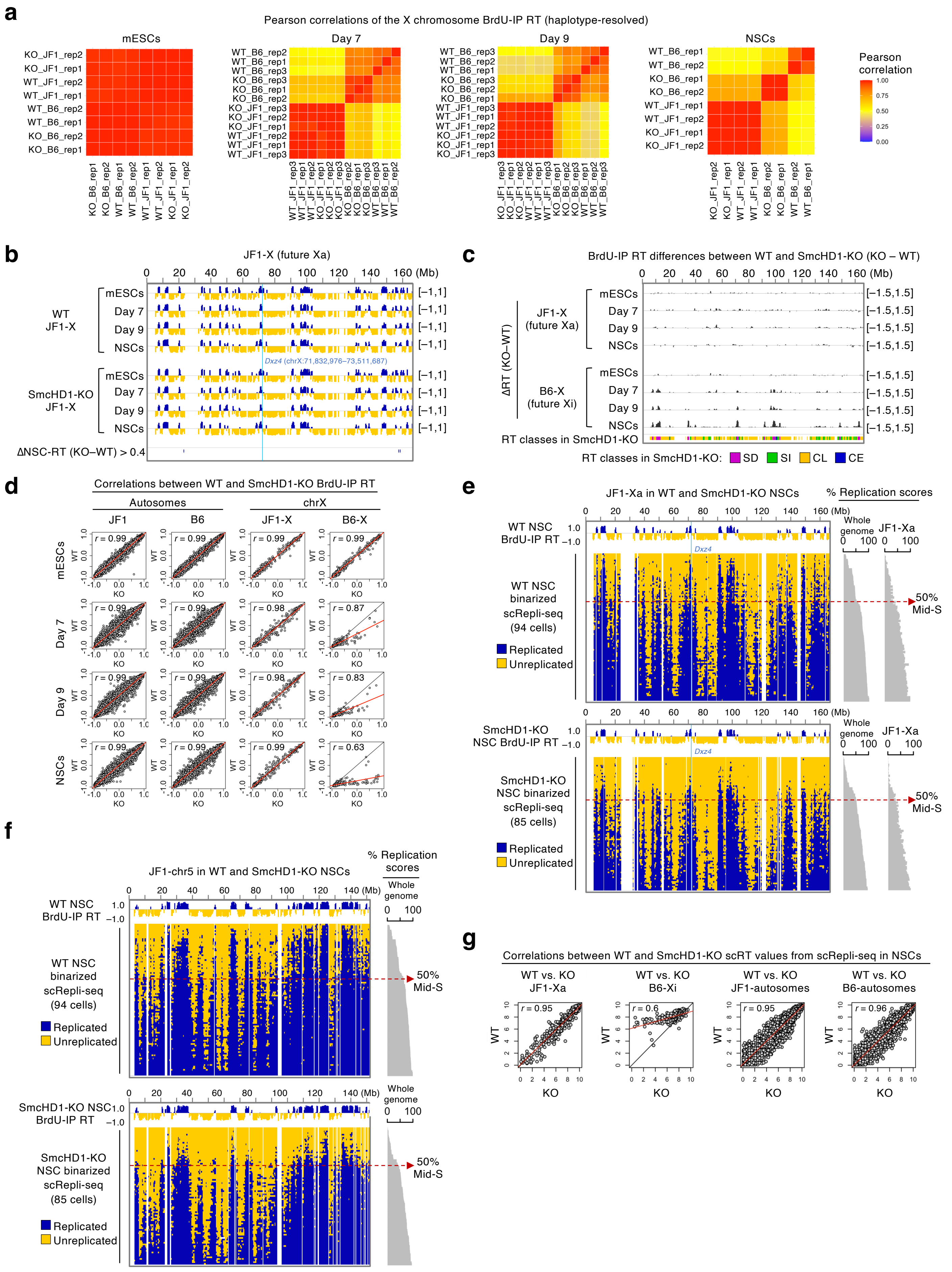

Supplementary Figure 3. BrdU-IP and scRepli-seq RT profile comparison of WT and SmcHD1-mutant cells during mESC differentiation

(**a**) Pearson correlations for pair-wise comparisons of haplotype-resolved JF1-X and B6-X BrdU-IP RT profiles (400-kb bins) of differentiation intermediates derived from WT and SmcHD1-mutant (KO) JB4/EI7HZ2 mESCs. RT profiles of the B6-Xi, which were relatively similar in WT and SmcHD1-mutant day 7 or 9 cells, were clearly different between WT and SmcHD1-mutant NSCs. (**b**) Haplotype-resolved BrdU-IP RT profiles of the JF1-X (future Xa) during WT and SmcHD1-mutant (KO) JB4/EI7HZ2 mESC differentiation (average of two or three biological replicates, 400-kb bins). Domains with RT differences of > 0.4 between the JF1-Xa in WT and SmcHD1-mutant (KO) NSCs are shown at the bottom. Blue line represents *Dxz4* position. (**c**) BrdU-IP RT differences between WT and SmcHD1-mutant (KO) cells during mESC differentiation. We subtracted haplotype-resolved BrdU-IP RT profile of WT cells from that of SmcHD1-mutant cells. While the RT profiles of the JF1-Xa did not show differences between WT and SmcHD1-mutant cells, those of the B6-Xi in day 7 9 cells and NSCs showed small and large differences, respectively. (**d**) Haplotype-resolved BrdU-IP RT values for each genomic bin of WT and SmcHD1-mutant (KO) cells were compared (400-kb bins). Pearson correlations (*r)* are shown. While high correlations were observed between WT and SmcHD1-mutant cells for the autosomes and the JF1-Xa regardless of the differentiation states, the B6-X showed high, moderate, and low correlations in mESCs, day 7 or 9, and NSCs, respectively. (**e,f**) Binarized whole-S scRepli-seq profiles of the JF1-Xa (e) and JF1-chr5 (f) in WT and SmcHD1-mutant (KO) NSCs throughout the S phase, as in Supplementary Figure 2d. RT profiles of the JF1-Xa (e), the JF1-chr5 (f), and the B6-chr5 (not shown) were similar between WT and SmcHD1-mutant NSCs. Blue line on chromosome X profiles represents *Dxz4* position. (**g**) Comparison of single-cell RT (scRT; defined as an estimated RT of a given genomic bin calculated from whole-S scRepli-seq data assuming a 10 h S-phase; see Methods) values of WT and SmcHD1-mutant (KO) NSCs. Pearson correlations (*r)* are shown. While the scRT values were similar between WT and mutant cells for the JF1-Xa and the autosomes, they were not for the B6-Xi.

Supplementary Figure 4

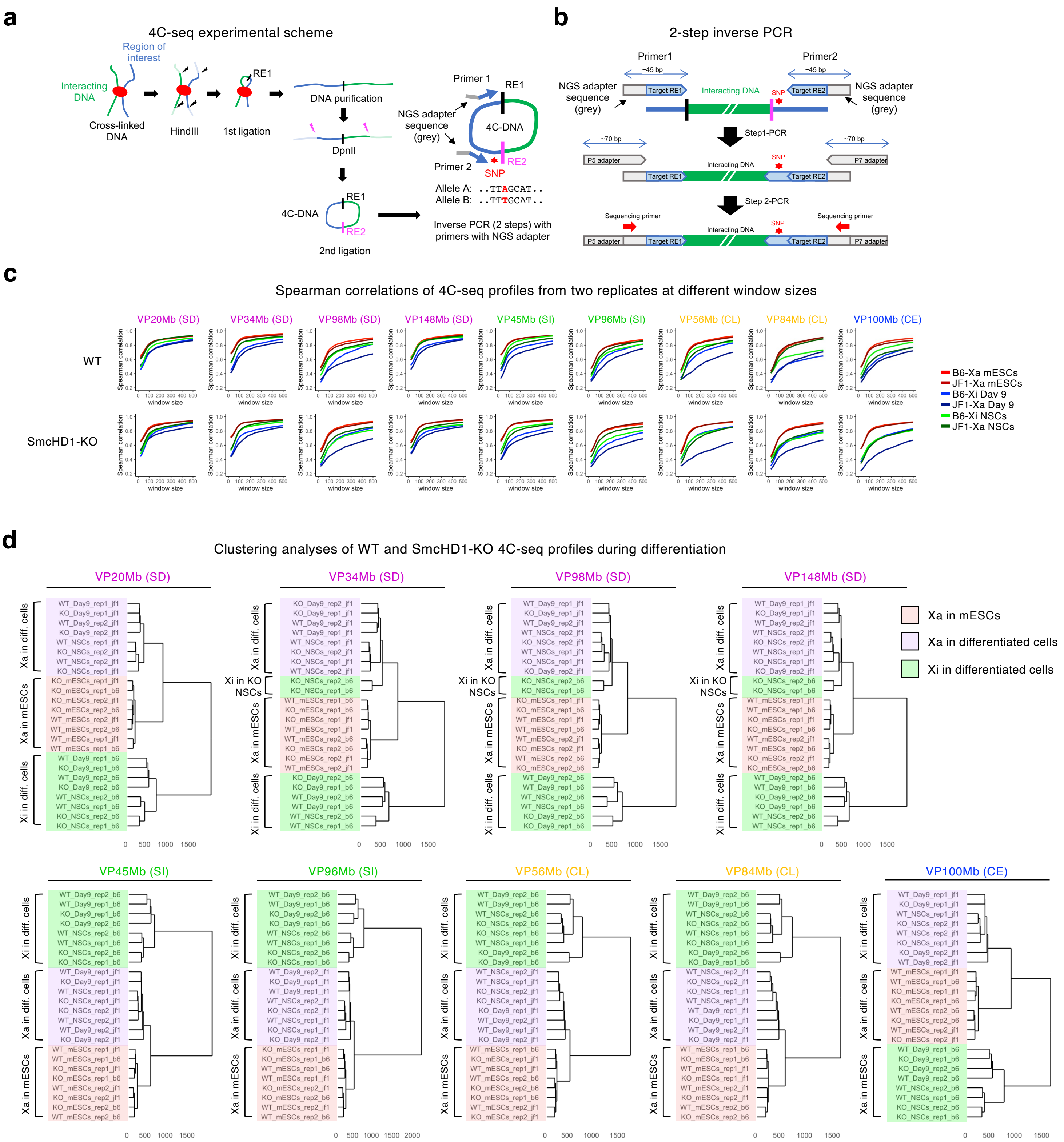

Supplementary Figure 4. 4C-seq profiling and clustering analysis of chromosome X during mESC differentiation

(**a**) Cells were fixed with 1% formaldehyde to stabilize chromatin organization within the nuclei. Crosslinked DNA was fragmented by a 6-base cutter HindIII followed by ligation, producing chimeric DNA molecules including those consisting of a region of interest (i.e., viewpoint, in blue) and its interacting DNA (in green). After DNA purification, chimeric DNA was trimmed by a 4-base cutter DpnII to generate smaller size fragments. Another round of ligation in a diluted condition causes self ligation, forming what we call 4C-DNA. The unknown interacting DNA fragments were amplified by inverse PCR using two primers 1 and 2 complementary to the ends of a viewpoint facing outward, which contained Illumina NGS adapter sequences and allowed the products to be directly sequenced by NGS (see details in the figure). To distinguish the two homologs, we designed primer 2 just upstream (<100 bp) of a region on the viewpoint that contains SNP(s). (**b**) To amplify the 4C-seq library by inverse PCR, primary primers 1 and 2 were used to amplify the ligated interacting DNA for several cycles (step-1). The step-1 PCR products were purified and used as templates for the second round PCR using universal primers that contained the remaining sequences of the Illumina NGS adapter including the index sequences (step-2). The final PCR products contained full-length Illumina NGS adapter sequences, allowing NGS directly from a 4C-seq library. (**c**) Spearman correlations of smoothed 4C-seq profiles between two replicates, which were calculated using several window sizes (sliding windows of 20, 100, 200, 250, and 500 fragments). (**d**) Comparison of allele-specific smoothed 4C-seq profiles of the X chromosomes in WT and SmcHD1-mutant (KO) mESCs, day 9, and NSCs, by hierarchical clustering. The 4C-seq profiles of the Xa are similar and cluster together regardless of SmcHD1 genotype or differentiation states (highlighted in pink and purple). As for the 4C-seq profiles of the B6-Xi in day 9 and NSCs (highlighted in green), the day-9 B6-Xi clustered together with the WT NSC B6-Xi for all viewpoints tested. As for the non-SD viewpoints (SI, CL, CE), the SmcHD1-mutant B6-Xi in day 9 and NSCs clustered together with the WT cells (in green). By contrast, for three out of four SD viewpoints (VP34Mb, VP98Mb, and VP148Mb), the SmcHD1-mutant NSC B6-Xi profiles separated from the WT counterpart and clustered together with the JF1-Xa in differentiated cells (day 9 and NSCs), suggesting changes in the SD domain contact profiles on the Xi in SmcHD1-mutant NSCs.

Supplementary Figure 5

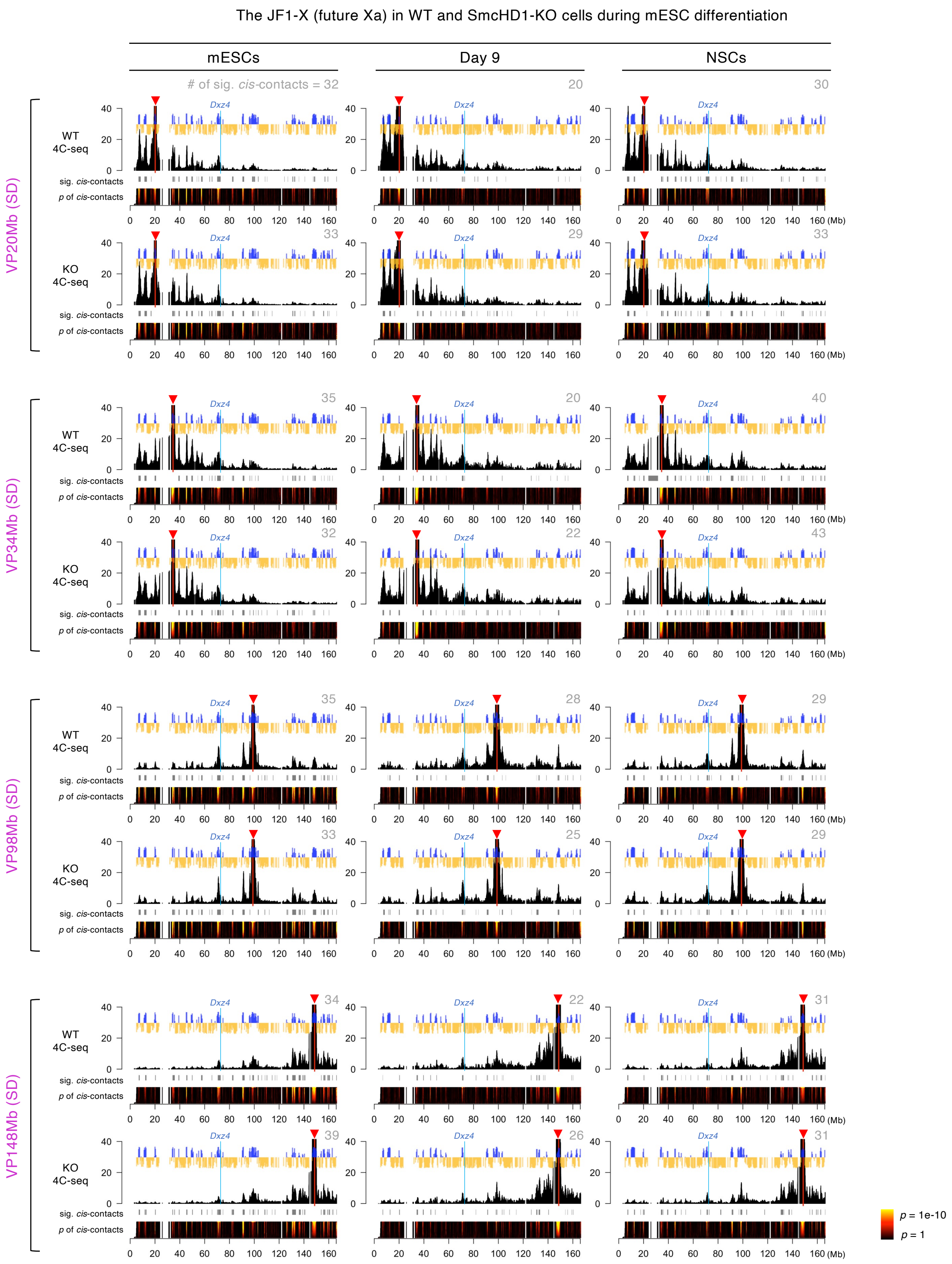

Supplementary Figure 5 (continue)

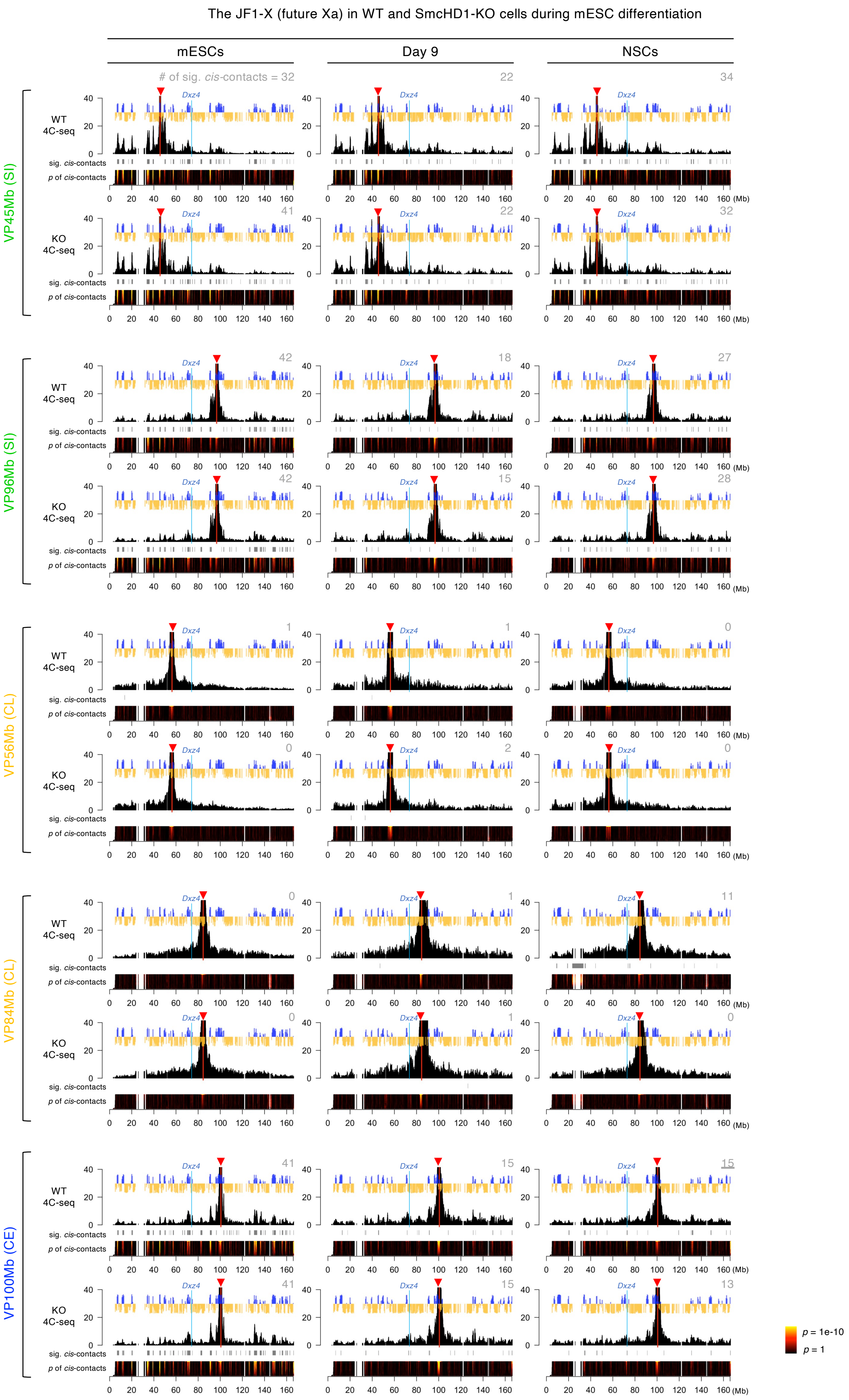

Supplementary Figure 5. 4C-seq profiles of the JF1-X during mESC differentiation

Smoothed 4C-seq profiles of the JF1-X (future Xa) in WT and SmcHD1-mutant (KO) cells during mESC differentiation. Reads from two replicates were combined, plotted, (in sliding windows of 201 restriction fragments, shown in black; see Methods), and overlaid on the BrdU-IP RT profiles (blue, early and yellow, late) for viewpoints (red lines and arrowheads) on the JF1-X. Blue lines, *Dxz4*; Gray bars beneath each plot, significant far-*cis* contacts (the number of such contacts is shown in the top right corner of each 4C plot in gray); Domainogram beneath each plot, the significance of the interaction shown by the color range (window sizes = 2–200 from bottom-to-top).

Supplementary Figure 6

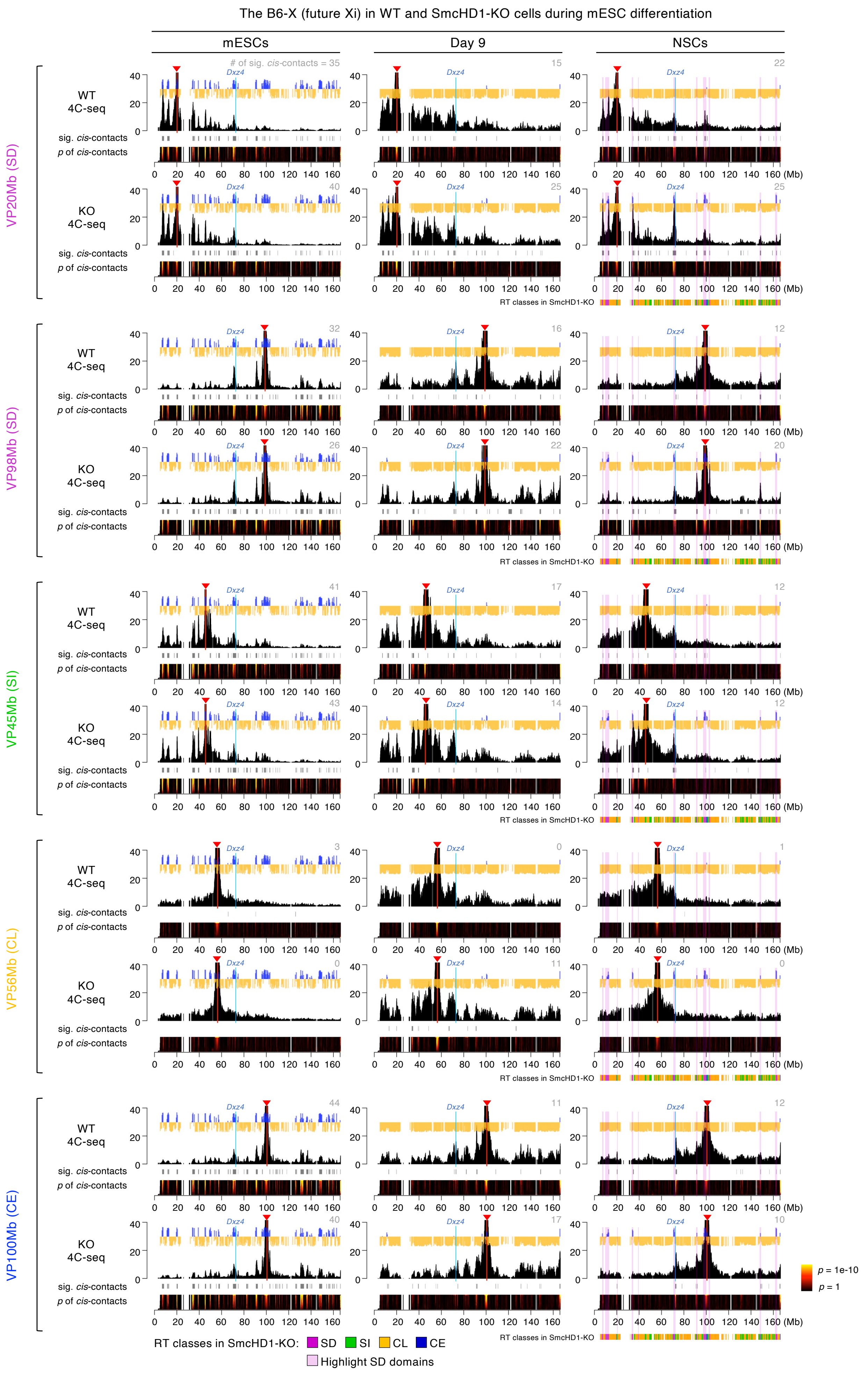

Supplementary Figure 6. 4C-seq profiles of the B6-X during mESC differentiation

Smoothed 4C-seq profiles of the B6-X (future Xi) in WT and SmcHD1-mutant (KO) cells during mESC differentiation. Reads from two replicates were combined, plotted (in sliding windows of 201 restriction fragments, shown in black; see Methods), and overlaid on the BrdU-IP RT profiles (blue, early and yellow, late) for viewpoints (red lines and arrowheads) on the B6-X. Blue lines, *Dxz4*; Gray bars beneath each plot, significant far-*cis* contacts (the number of such contacts is shown in the top right corner of each 4C plot in gray); Domainogram beneath each plot, the significance of the interaction shown by the color range (window sizes = 2–200 from bottom-to-top); Pink highlighted regions, SD domains; Colored bars, RT classes.

Supplementary Figure 7

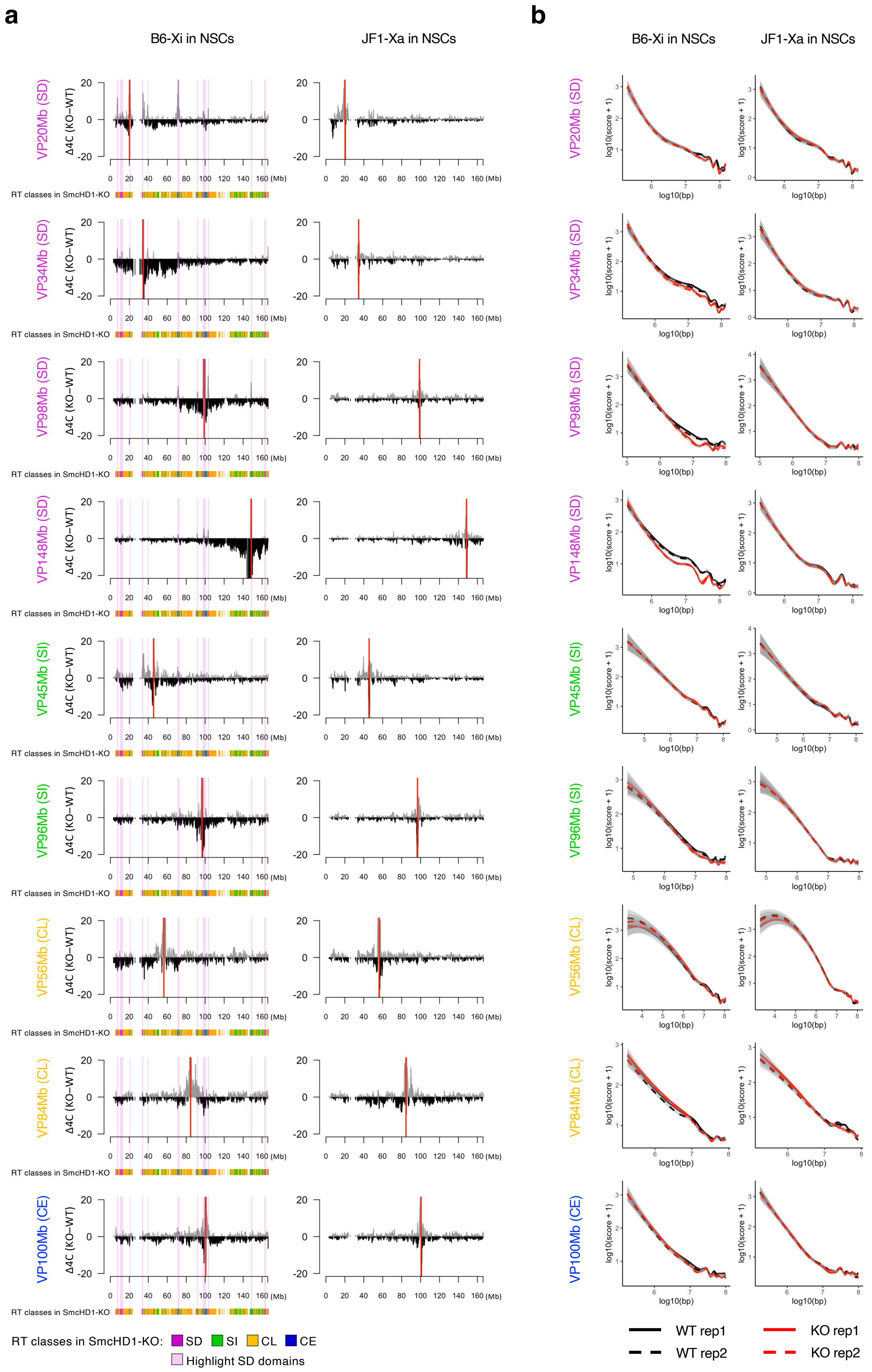

Supplementary Figure 7. Differences in 4C-seq profiles between WT and SmcHD1-mutant NSCs

(**a**) Differences in 4C-seq profiles between WT and SmcHD1-mutant (KO) NSCs (KO–WT). (**b**) Distance decay plots of individual 4C viewpoints. The grey band shows the 95% confidence interval for predictions from the loess model. A decrease in interaction frequency as a function of genomic distance was observed as expected. However, the decrease was more pronounced when analyzing the SD viewpoints on the SmcHD1-mutant NSC Xi, indicating that the SD domains exhibited weaker *cis* interactions. This is consistent with the idea that the SD domains protrude out of the Xi territory.

Supplementary Figure 8

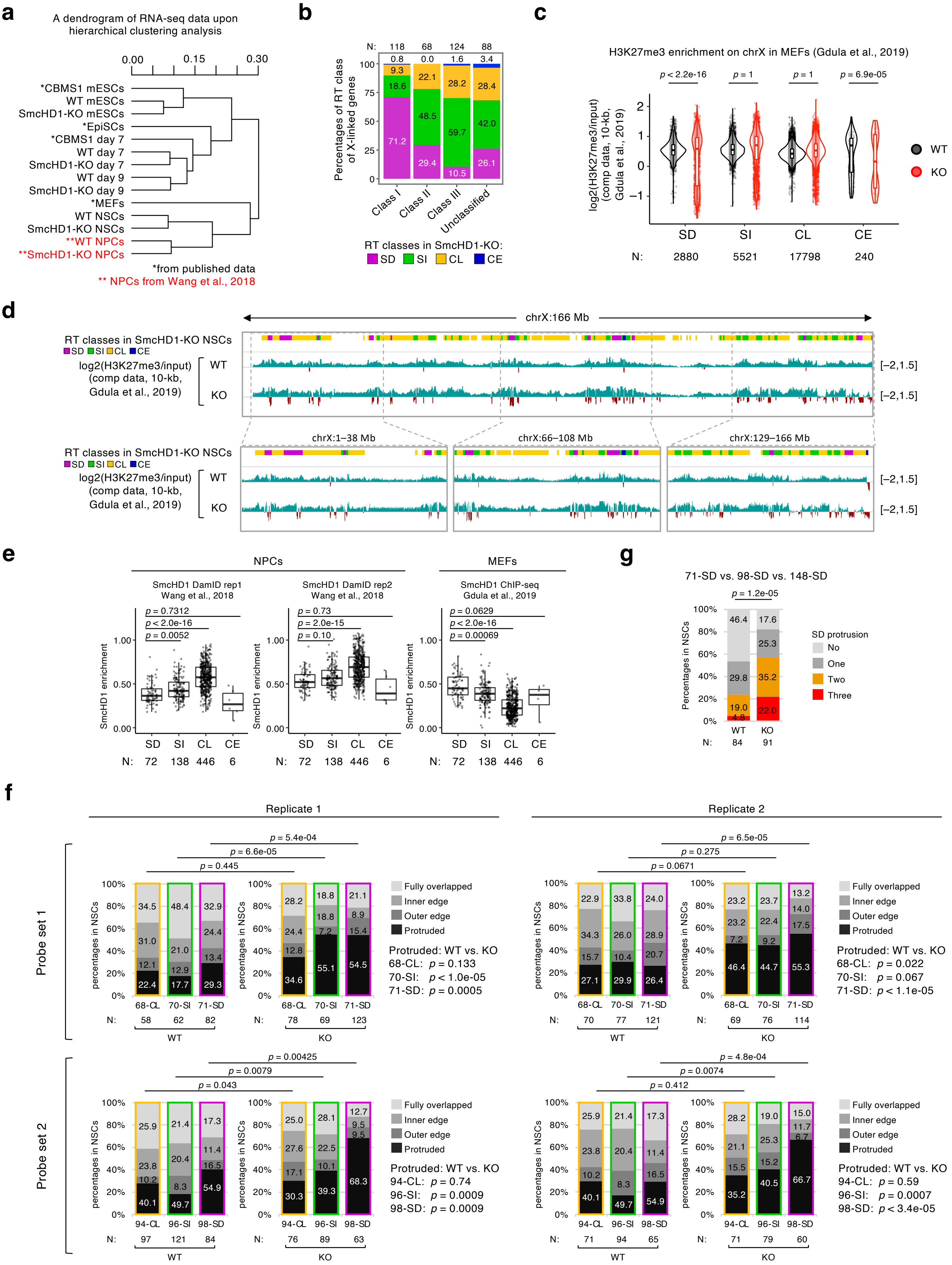

Supplementary Figure 8. Characteristics of the SD domains and their protrusion out of the Xi core as observed by FISH

(**a**) Comparison of RNA-seq profiles of our NSCs with NPCs from Wang et al[2]. (red) by hierarchical clustering reveals their similarity. RNA-seq profiles of various differentiation intermediates were also included as in Supplementary Figure 1e. (**b**) Class I (SmcHD1-sensitive), Class II (partially SmcHD1-sensitive), Class III (SmcHD1-insensitive), and Unclassified X-linked genes identified by Wang et al.[2] and their relationship to our RT classes. N, the number of X-linked genes in each class. (**c**) Comparison of H3K27me3 enrichment in different RT domains in the WT (black) and SmcHD1-mutant (KO) (red) MEFs from Gdula et al[3]. The log2[IP/input] of non-haplotype resolved data is shown (10-kb). In SmcHD1-mutant MEFs, the loss of H3K27me3 was observed in the SD domains, and to a lesser extent, in the SI domains. *P*-values were obtained from the one-sided Wilcoxon signed-rank test with an alternative hypothesis of the WT to have a higher H3K27me3 than SmcHD1-mutant MEFs. The data from merged replicates was used. N, the number of genomic bins in each class. (**d**) H3K27me3 ChIP-seq profiles on chromosome X in MEFs[3]. Upper and lower panels show the entire Xi and the magnified views of three representative regions, respectively. Colored bars, RT classes. (**e**) Enrichment of SmcHD1 in different RT domains in NPCs and MEFs, as assayed by ScmHD1 Dam ID[2] and SmcHD1 ChIP-seq[3]. The non-haplotype resolved data is shown (200-kb). *P*-values were obtained from two-sided Wilcoxon signed-rank test with Bonferroni correction. N, the number of genomic bins in each class. (**f**) Percentages of DNA-FISH signal localization of DNA-FISH probes (as shown in Figure 5c,e) relative to the *Xist* cloud in NSCs for each biological replicate. N, the total number of cells analyzed from each replicate. *P*-values were obtained from a chi-square test for all groups and a Fisher's exact test for the protruded group. (**g**) Percentages of the localization patterns of three SD probes (71-SD, 98-SD, and 148-SD; as shown in Figure 5g) in WT and SmcHD1-mutant (KO) NSCs. Simultaneous protrusion of two and three SD probes was much more frequent in SmcHD1-mutant NSCs than in WT NSCs. N, the total number of cells analyzed from three independent replicates. *P*-values were obtained from a chi-square test for all groups.

Supplementary Figure 9

a

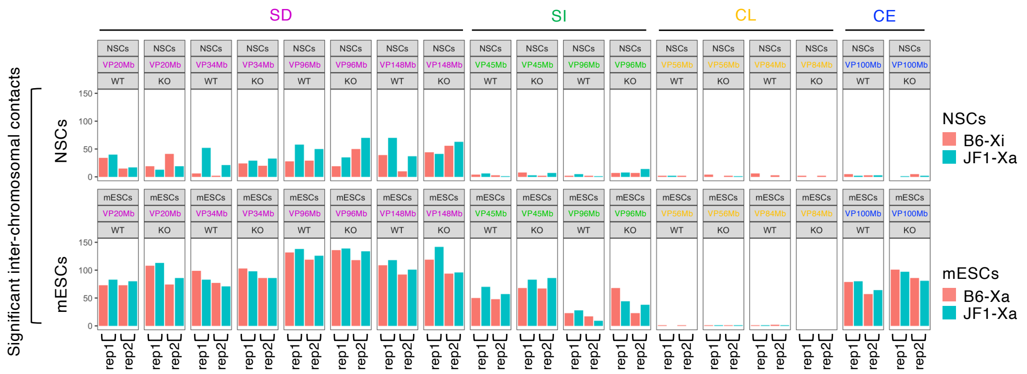

b

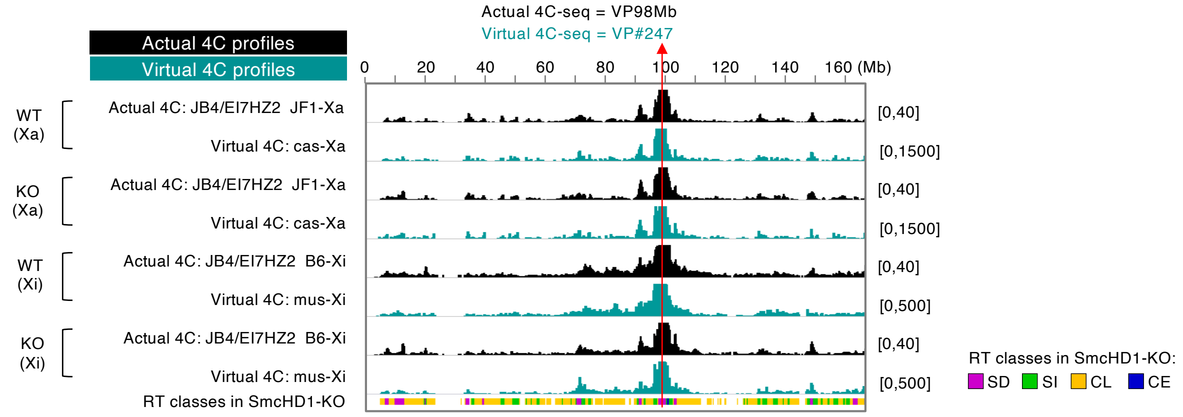

c

RT class in SmcHD1-KO NSCs	Bins (400-kb)	Total (Mb)	Number of Refseq annotated genes (mm9)	Number of escapees in NPCs by Giorgetti et al., 2016	Number of escapees in Patski cells by Berletch et al., 2015	Number of escapees in tissues by Berletch et al., 2015	Number of escapees in XCI-induced mESCs for 24 hr by Barros de Andrade E Sousa et al., 2019
SD	36	14.4	266	55	24	14	26
SI	69	27.6	311	17	17	12	7
CL	223	89.2	339	6	10	11	3
CE	3	1.2	15	1	2	3	3

d

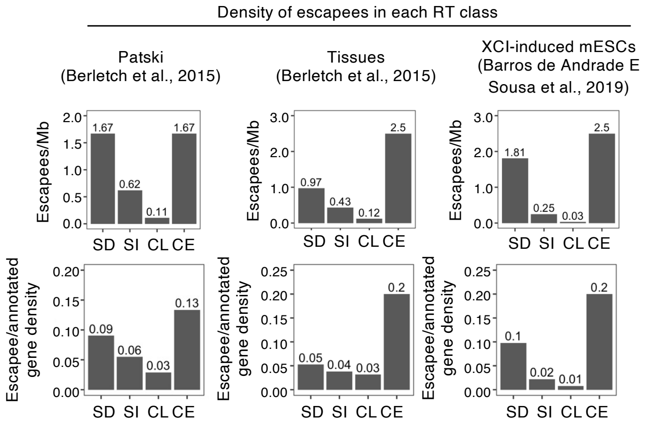

e

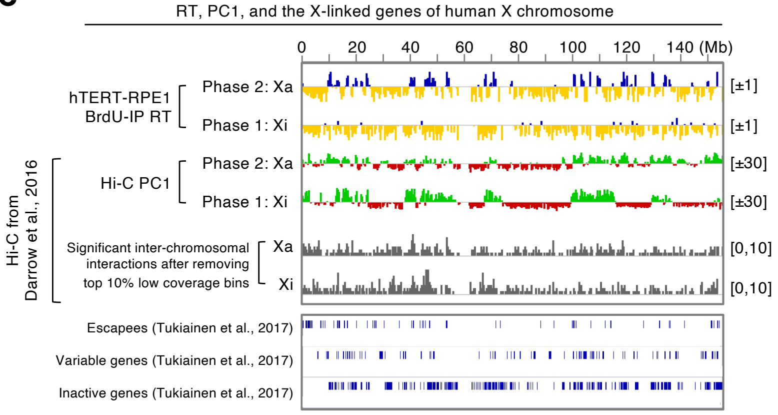

f

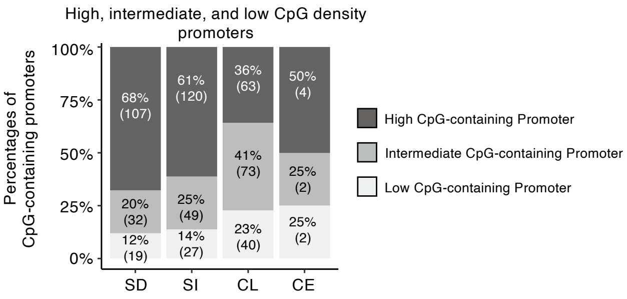

Supplementary Figure 9. Analyses of interchromosomal contacts, virtual 4C-seq, mouse escapee gene density, promoter CpG density, and human TERT-RPE1 RT/Hi-C/virtual 4C data

(**a**) 4C-seq profiles of WT and SmcHD1-mutant (KO) NSCs were analyzed for significant interchromosomal interactions using Splinter et al.'s pipeline[4]. Results from two replicates are shown. In NSCs, significant interchromosomal interactions were frequently observed for the SD viewpoints but not the SI, CL, and CE viewpoints on both the JF1-Xa and the B6-Xi. In mESCs, significant interchromosomal interactions were also frequently observed for the SD but not CL viewpoints. However, an SI (VP45Mb) and a CE viewpoint also showed frequent interchromosomal interactions, comparable to those of the SD viewpoints. (**b**) A comparison of actual 4C-seq profiles derived from VP98Mb (SD) and virtual 4C-seq profiles derived from virtual VP#247 (corresponding to chrX:98,400,000–98,800,000), which are from the same region. Red line, viewpoint position. (**c**) Numbers of annotated genes (mm9) and escapees[5–7] in the SD, SI, CL, and CE domains. (**d**) Mouse escapee density in different RT domains based on two studies[6,7]. (**e**) Allele-specific BrdU-IP RT profiling of human TERT-RPE1 (hTERT-RPE1) cells using our in-house SNP information of the corresponding hTERT-RPE1 line used. Phases 1 and 2 represent alleles for the late-replicating Xi and the early-replicating Xa, respectively. Using the SNP information, we re-analyzed hTERT-RPE1 Hi-C data[8] by performing PC analysis for each phase by HOMER[9] using default parameters. Hi-C PC1 revealed that phases 1 and 2 represented the Xi and the Xa, respectively, consistent with RT data. Therefore, we further created virtual 4C-seq profiles of hTERT-RPE1 Xa (phase 2) and the Xi (phase 1) from 388 viewpoints (400-kb bins). Significant interchromosomal interactions for each virtual viewpoint were counted and plotted along the human X chromosome (gray profiles). Inactive genes, variable genes, and escapees[10] are shown for comparison. (**f**) Percentages of high, intermediate, and low CpG-containing promoters in different RT domains. The number within parentheses indicates the number of X-linked gene promoters. A list of CpG-containing promoters from Mikkelsen et al[11] was used for the analysis.

Supplementary Figure 10

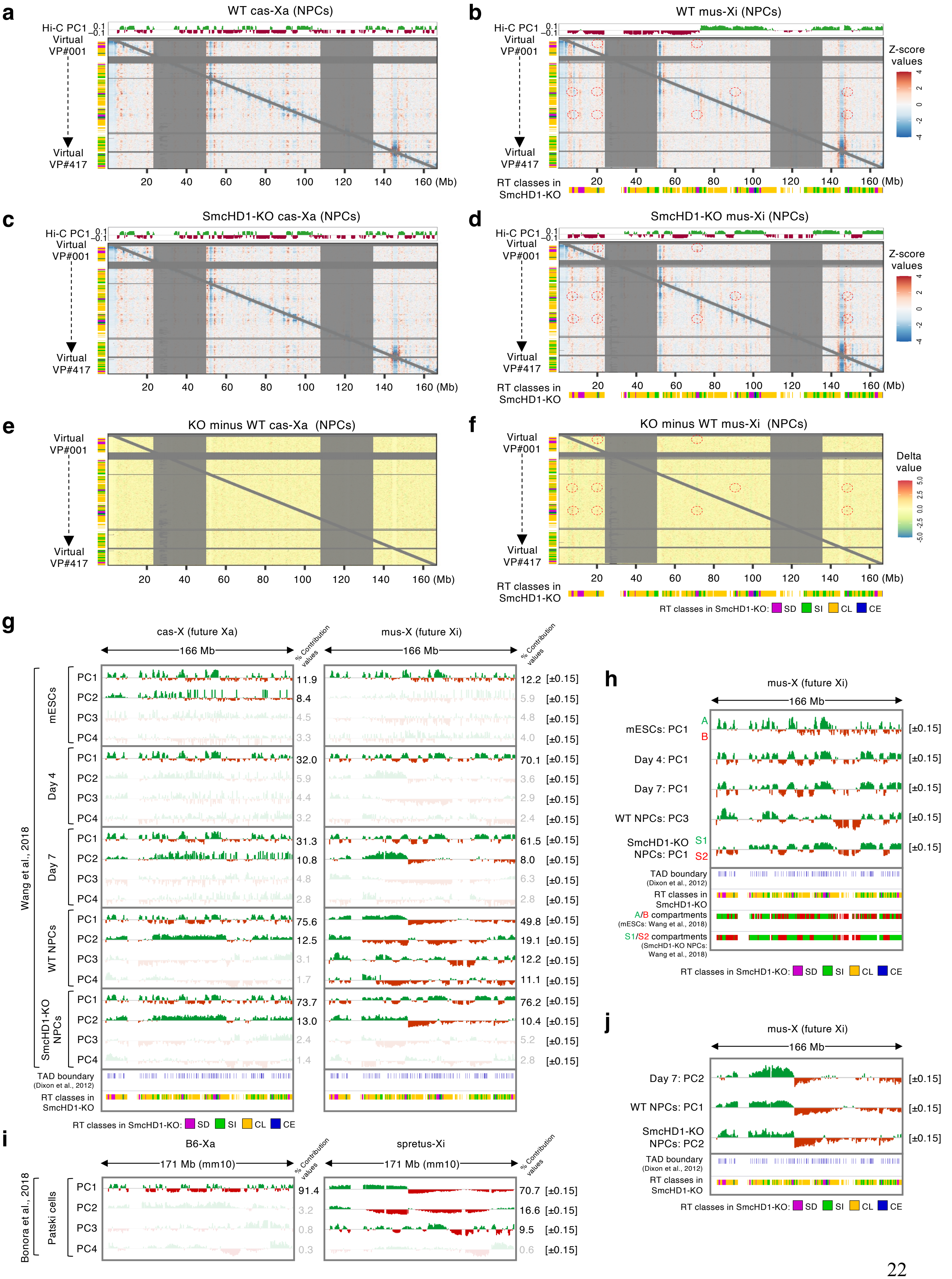

Supplementary Figure 10. Z-score heatmaps and PC1–4 of the mouse Xi Hi-C data

(**a**–**d**) Virtual 4C analysis and z-score conversion of Hi-C data. X chromosomes (cas-Xa, mus-Xi) in WT and SmcHD1-mutant (KO) NPCs are shown. Normalized reads from virtual 4C (5-kb bins) were made binary and the relative enrichment (z-score) was calculated using sliding windows of 250 kb (=5-kb × 50). Average z-scores of nonoverlapping 400-kb bins are plotted as heatmaps. High z-scores represent strong interactions. RT classes are shown along the vertical and horizontal axes. (**e**,**f**) Subtraction plots (KO–WT) of z-score heatmaps of the cas-Xa and mus-Xi. Gray areas represent low-coverage areas. (**g**) Published Hi-C data were reanalyzed at 500-kb resolution (mm9)[2]. We calculated PC1–4 on the Xa and the Xi of various differentiation states and genotypes (WT or KO) and plotted those with >8% contribution rates. TAD boundary positions in mESCs[12] and RT classes are shown. (**h**) Supplementary Figure 10g revealed that the PC3 of WT NPC mus-Xi looked very similar to the PC1 (S1/S2 compartments) of the SmcHD1-mutant (KO) NPC mus-Xi, as well as the PC1 (A/B compartments) of the WT mESC mus-Xa (and the cas-Xa), which resembled day-4 and day-7 PC1 profiles. TAD boundary positions in mESCs[12], RT classes, binarized A/B compartments[2], and binarized S1/S2 compartments[2] are shown. (**i**) The same analysis as in Supplementary Figure 10g using allele-specific Hi-C data (mm10) of mouse Patski cells[13], which were fibroblast cells derived from the embryonic kidney. PC1–4 of mouse Patski cells were similar to those of WT NPCs (Supplementary Fig. 10g), suggesting similar Xi structure in different cell lineages. (**j**) Based on Supplementary Figure 10g, we found that a PC corresponding to the megadomain structure separation was observed only upon differentiation. TAD boundary positions in mESCs[12] and RT classes are shown.

Supplementary Texts

Supplementary Text 1

Cell-to-cell heterogeneity of SD domain protrusion in SmcHD1-mutant NSCs

Our 4C-seq results and FISH analysis suggested that the SD domains protruded out of the Xi within the same cell in SmcHD1-mutant NSCs (Figs. 3b,5 and Supplementary Fig. 6). Pair-wise analysis revealed that the protrusion of the SD domains is frequent, with some cells exhibiting two SD protrusions while others exhibit only one (Fig. 5i). When we further analyzed three SD probes within the same cell, we observed an even higher frequency of simultaneous protrusion of multiple SD probes in SmcHD1-mutant NSCs (Supplementary Fig. 8g). It is likely that the more SD probes we analyze, the more frequent the simultaneous protrusion of multiple SD probes would be, as the probability increases. However, the variation of protrusion patterns observed here suggests cell-to-cell heterogeneity in SD domain protrusion.

Because scRepli-seq showed RT reversal of SD domains in most SmcHD1-mutant NSCs (Fig. 2d), it is unlikely that the cell-to-cell heterogeneity in SD domain protrusion is derived from cell-to-cell RT heterogeneity. Since the degree of RT reversal varied for each SD domain (i.e., some domains became earlier replicating than others), it is possible that some SD domains might have a greater capability to protrude than others, as RT reflects compartmentalization. This difference in protrusion might affect how they are captured by FISH analysis, which is less sensitive than the NGS-based approach. However, the heterogeneity observed might also be due to the limitation of FISH experiments, which can only capture a snapshot of chromatin dynamics.

Supplementary Text 2

SD-SD interactions in SmcHD1-mutant NSCs

The 71-SD and 98-SD probes that simultaneously protruded out of the Xi core (referred to as two-SD protrusion) showed a closer distance between each other in SmcHD1-mutant NSCs compared to WT NSCs (Fig. 5j, pair-1), which was consistent with their interaction frequency based on 4C-seq data (Fig. 5g, pair-1). However, even when only one (one-SD protrusion) or none (no-SD protrusion) of these two SD probes protruded out in SmcHD1-mutant NSCs, the probe-to-probe distance was similarly close to when they simultaneously protruded out (Fig. 5k, pair-1 in KO, the three lines closely overlapped and didn't show significant differences). Thus, it is possible that SD domains also interact with each other inside the *Xist* cloud in SmcHD1-mutant NSCs. However, it should be noted that when only one or none of these two SD probes protruded out, the probe-to-probe distance was similarly close between WT and SmcHD1-mutant NSCs (Fig. 5j, pair-1). Thus, while it is possible that SD domains could contact each other inside the *Xist* cloud in mutant NSCs, such interaction would not result in decreased probe-to-probe distance, likely due to the resolution limit of the assay. This could be an alternative explanation for why we did not observe closer probe-to-probe distance in SmcHD1-mutant NSCs for probe pairs 2 and 3.

Supplementary Text 3

Characteristics of CE domains

CE domains are always early replicating on the Xi in NSCs, regardless of the SmcHD1-genotype. scRepli-seq also confirmed that they replicate in the first half of the S-phase, suggesting the robustness of their RT property (Fig. 2b–c). While CE domains have lower escapee density than the SD domains in NPCs (Fig. 7a), they have more escapees than the SD domains in other cell types (Supplementary Fig. 9c–d). This suggests that CE domains are also unstable regions and relatively prone to be reactivated. Moreover, CE domains also contain well-known escapees, such as *Xist*, which is highly expressed on the Xi, and *Mid1*, which carries a portion of pseudoautosomal regions[14]. These may explain why these domains are consistently maintained as active early replicating on the Xi.

Description of Supplementary Tables

Supplementary Table 1. Genomic coordinates of the SI, SD, CL and CI domains (mm9)

Supplementary Table 2. List of sequencing experiments

Supplementary Table 3. Percentage replication scores of scRepli-seq in wild-type and SmcHD1-mutant NSCs

Supplementary Table 4. List of primers and sequences used in 4C-seq experiments

Supplementary Table 5. Sequences used to identify reads from B6 or JF1 alleles in 4C-seq data

Supplementary Table 6. 4C-seq data quality metrics

Supplementary Table 7. List of escapees in mice used in this study

Supplementary References

1. Miura, H. *et al.* Single-cell DNA replication profiling identifies spatiotemporal developmental dynamics of chromosome organization. *Nat. Genet.* **51**, 1356–1368 (2019).
2. Wang, C.-Y., Jégu, T., Chu, H.-P., Oh, H. J. & Lee, J. T. SMCHD1 Merges Chromosome Compartments and Assists Formation of Super-Structures on the Inactive X. *Cell* **174**, 406-421.e25 (2018).
3. Gdula, M. R. *et al.* The non-canonical SMC protein SmcHD1 antagonises TAD formation and compartmentalisation on the inactive X chromosome. *Nat. Commun.* **10**, 30 (2019).
4. Splinter, E., de Wit, E., van de Werken, H. J. G., Klous, P. & de Laat, W. Determining long-range chromatin interactions for selected genomic sites using 4C-seq technology: from fixation to computation. *Methods* **58**, 221–230 (2012).
5. Giorgetti, L. *et al.* Structural organization of the inactive X chromosome in the mouse. *Nature* **535**, 575–579 (2016).
6. Berletch, J. B. *et al.* Escape from X inactivation varies in mouse tissues. *PLoS Genet.* **11**, e1005079 (2015).
7. Barros de Andrade E Sousa, L. *et al.* Kinetics of Xist-induced gene silencing can be predicted from combinations of epigenetic and genomic features. *Genome Res.* **29**, 1087–1099 (2019).
8. Darrow, E. M. *et al.* Deletion of DXZ4 on the human inactive X chromosome alters higher-order genome architecture. *Proc. Natl. Acad. Sci. U. S. A.* **113**, E4504-12 (2016).
9. Heinz, S. *et al.* Simple combinations of lineage-determining transcription factors prime cis-regulatory elements required for macrophage and B cell identities. *Mol. Cell* **38**, 576–589 (2010).
10. Tukiainen, T. *et al.* Landscape of X chromosome inactivation across human tissues. *Nature* **550**, 244–248 (2017).
11. Mikkelsen, T. S. *et al.* Genome-wide maps of chromatin state in pluripotent and lineage-committed cells. *Nature* **448**, 553–560 (2007).
12. Dixon, J. R. *et al.* Topological domains in mammalian genomes identified by analysis of chromatin interactions. *Nature* **485**, 376–380 (2012).
13. Bonora, G. *et al.* Orientation-dependent Dxz4 contacts shape the 3D structure of the

inactive X chromosome. *Nat. Commun.* **9**, 1445 (2018).

14. Dal Zotto, L. *et al.* The mouse Mid1 gene: implications for the pathogenesis of Opitz syndrome and the evolution of the mammalian pseudoautosomal region. *Hum. Mol. Genet.* **7**, 489–499 (1998).

Supplementary Data

Supplementary Data 1

For Supplementary Figure 1a

Marker = ϕX 174-*Hae* III digest

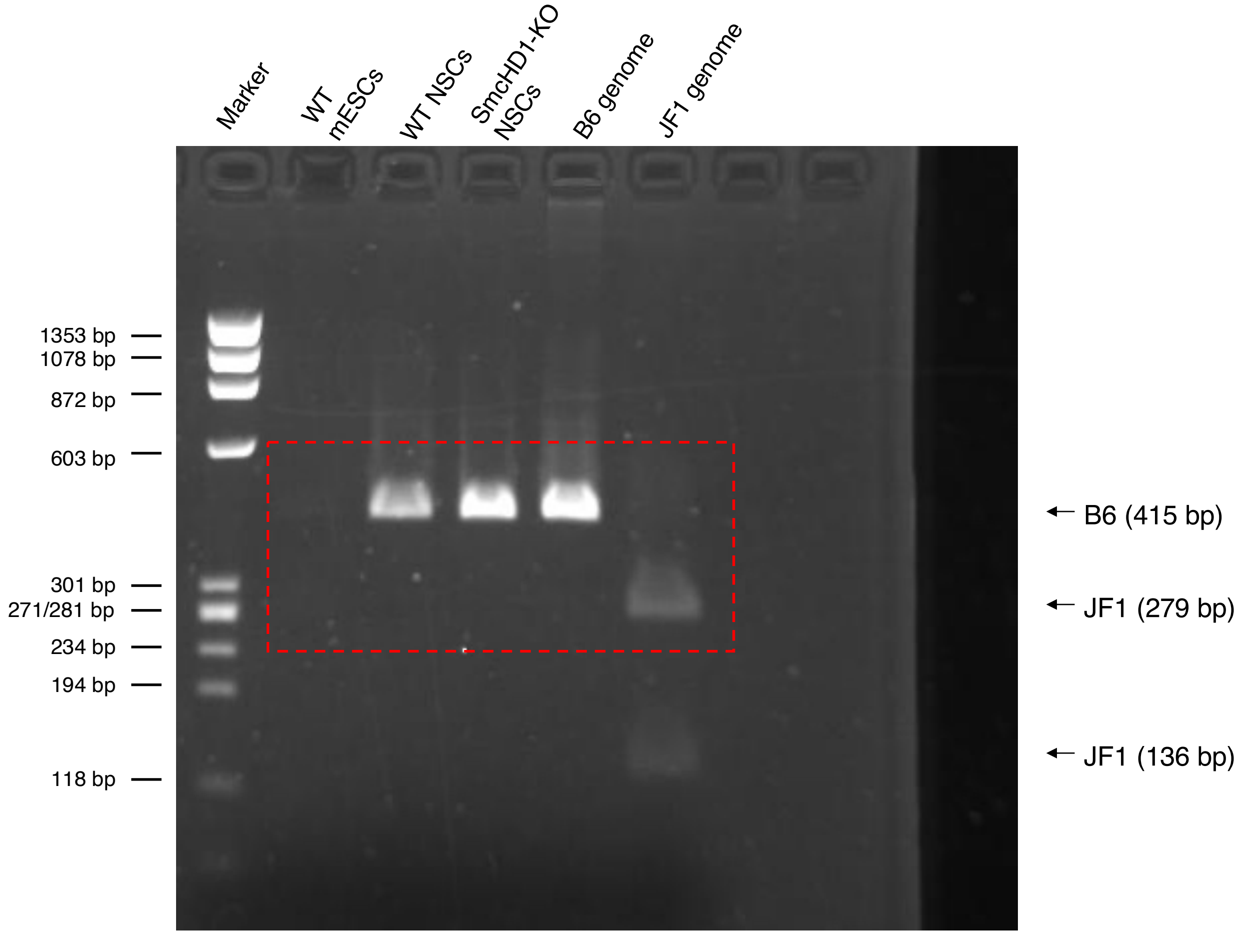

Supplementary Data 2

For Supplementary Figure 1b

Marker = ϕX 174-*Hae* III digest
(+) and (–) indicate the presence and absence of reverse transcriptase in the reaction, respectively

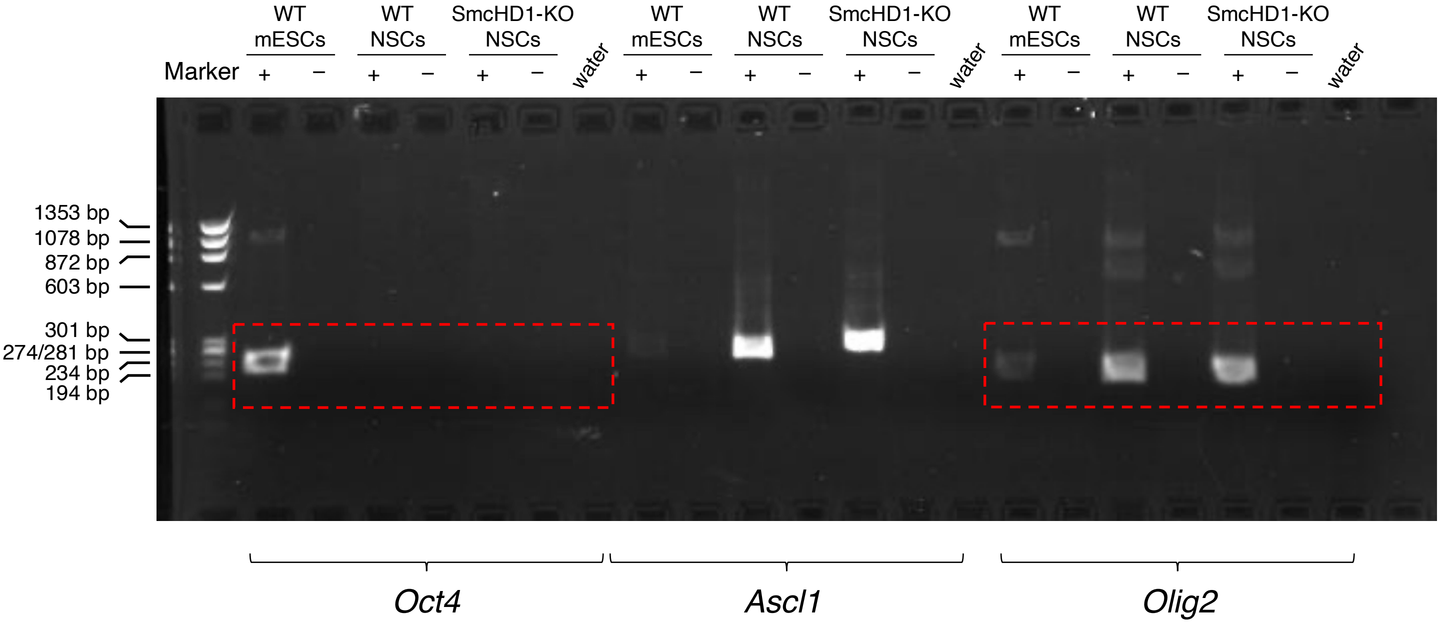

Marker = ϕX 174-*Hae* III digest

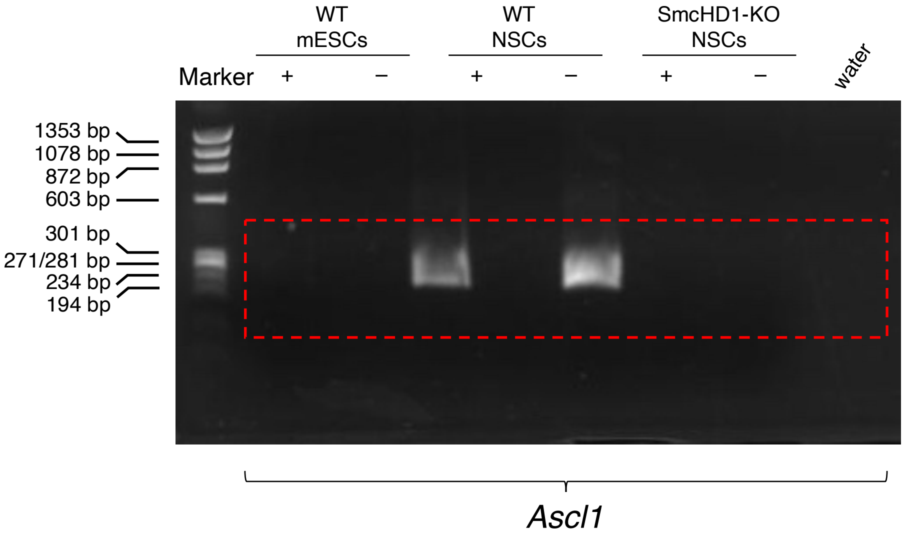

Supplementary Data 3

For Supplementary Figure 1h

Biorad Protein Dual color
standard cat# 161-0374

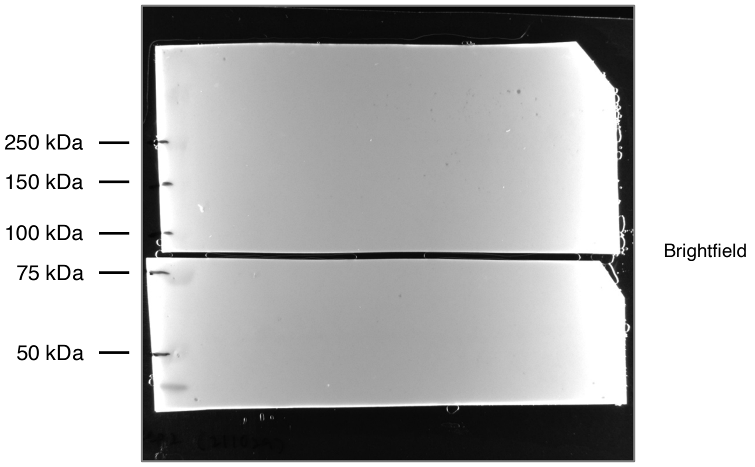

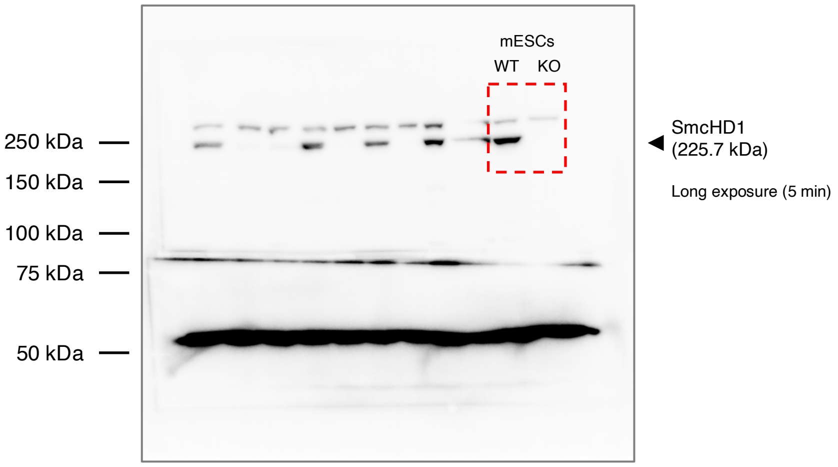

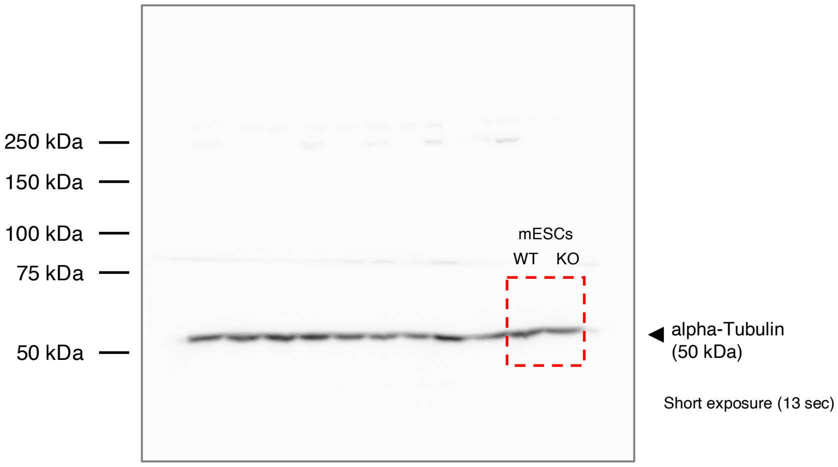

Supplement: Supplementary file 1 — Supplementary Figs. 1–10, Texts 1–3, Descriptions of Tables 1–7 and Data 1–3. [file 41594_2023_1052_MOESM1_ESM.pdf]
