# Supplementary material for: Replication dynamics identifies the folding principles of the inactive X chromosome
Source: Nat Struct Mol Biol. 2023 Aug 10;30(8):1224–37. doi: 10.1038/s41594-023-01052-1 (PMC10442229; doi:10.1038/s41594-023-01052-1)
Supplement: Supplementary file 2 — Reporting Summary [file 41594_2023_1052_MOESM2_ESM.pdf]

nature portfolio

Corresponding author(s): Ichiro Hiratani

Last updated by author(s): Jun 28, 2023

Reporting Summary

Nature Portfolio wishes to improve the reproducibility of the work that we publish. This form provides structure for consistency and transparency in reporting. For further information on Nature Portfolio policies, see our Editorial Policies and the Editorial Policy Checklist.

Statistics

For all statistical analyses, confirm that the following items are present in the figure legend, table legend, main text, or Methods section.

n/a	Confirmed	
☐	☒	The exact sample size (*n*) for each experimental group/condition, given as a discrete number and unit of measurement
☐	☒	A statement on whether measurements were taken from distinct samples or whether the same sample was measured repeatedly
☐	☒	The statistical test(s) used AND whether they are one- or two-sided<br>*Only common tests should be described solely by name; describe more complex techniques in the Methods section.*
☒	☐	A description of all covariates tested
☐	☒	A description of any assumptions or corrections, such as tests of normality and adjustment for multiple comparisons
☐	☒	A full description of the statistical parameters including central tendency (e.g. means) or other basic estimates (e.g. regression coefficient) AND variation (e.g. standard deviation) or associated estimates of uncertainty (e.g. confidence intervals)
☐	☒	For null hypothesis testing, the test statistic (e.g. *F*, *t*, *r*) with confidence intervals, effect sizes, degrees of freedom and *P* value noted<br>*Give P values as exact values whenever suitable.*
☒	☐	For Bayesian analysis, information on the choice of priors and Markov chain Monte Carlo settings
☒	☐	For hierarchical and complex designs, identification of the appropriate level for tests and full reporting of outcomes
☐	☒	Estimates of effect sizes (e.g. Cohen's *d*, Pearson's *r*), indicating how they were calculated

Our web collection on statistics for biologists contains articles on many of the points above.

Software and code

Policy information about availability of computer code

Data collection

Images were collected by DeltaVision Olympus IX71 inverted microscope and a standard SoftWoRx acquisition software version 6.5.2.

Data analysis

We used custom codes from Takahashi et al., Nature Genetics 2019 and Miura et al., Nature Protocols 2020 for BrdU-IP Repli-seq and single-cell Repli-seq (scRepli-seq) analyses. These custom codes are available at https://github.com/kuzobuta/hic_paper_NG_2019.
We used R pipelines from Krijger et al., Methods 2020 for 4C-seq analyses.
We used R pipelines from Splinter et al, Methods 2012 to analyze significant cis- and trans-interactions of 4C-seq data.
We used tophat2 version 2.1.1, (Kim et al., 2013), Cufflinks (Trapnell et al., 2010) and CummeRbund (Trapnell et al., 2012) for RNA-seq analyses.
We used Fiji (Schindelin, J et al. 2012) for imaging analyses. We used TurboReg plugin (Thévenaz et al., 1998) for coordinate correction of images. A standard Find Maxima and Mean Shift plugins in Fiji were used.
We used HOMER (Heinz et al., 2010) to generate virtual 4C-seq profiles from Hi-C data.
We used cooler (Abdennur et al., 2020) for Hi-C analyses and Genova (Van Der Weide et al., 2021) to plot Hi-C heatmaps.
We also used the following programs for NGS analyses: trim_galore version 0.6.6, BWA version v.0.7.17-r1188 (Li H et al., 2010), SAMtools (Li et al., 2009), BEDTools (Quinlan and Hall, 2010), Cutadapt (M Martin, 2010), FastQC (http://www.bioinformatics.babraham.ac.uk /projects/fastqc/), Picard (http://broadinstitute.github.io/picard), Liftover (http://hgdownload.soe.ucsc.edu/admin/exe/), AneuFinder (R package, 1.2.1).
Cell Sorter Software version 2.1.6 was used for SONY SH800 flow cytometry.

For manuscripts utilizing custom algorithms or software that are central to the research but not yet described in published literature, software must be made available to editors and reviewers. We strongly encourage code deposition in a community repository (e.g. GitHub). See the Nature Portfolio guidelines for submitting code & software for further information.

Data

Policy information about availability of data

All manuscripts must include a data availability statement. This statement should provide the following information, where applicable:

- Accession codes, unique identifiers, or web links for publicly available datasets
- A description of any restrictions on data availability
- For clinical datasets or third party data, please ensure that the statement adheres to our policy

All replication timing datasets (BrdU-IP and scRepli-seq), 4C-seq, and RNA-seq datasets have been deposited in Gene Expression Omnibus under accession GSE211574.

Human research participants

Policy information about studies involving human research participants and Sex and Gender in Research.

Reporting on sex and gender	Human research is not involved in this study.
Population characteristics	Human research is not involved in this study.
Recruitment	Human research is not involved in this study.
Ethics oversight	Human research is not involved in this study.

Note that full information on the approval of the study protocol must also be provided in the manuscript.

Field-specific reporting

Please select the one below that is the best fit for your research. If you are not sure, read the appropriate sections before making your selection.

☒ Life sciences ☐ Behavioural & social sciences ☐ Ecological, evolutionary & environmental sciences

For a reference copy of the document with all sections, see nature.com/documents/nr-reporting-summary-flat.pdf

Life sciences study design

All studies must disclose on these points even when the disclosure is negative.

Sample size	No sample-size calculation was performed. The sample size was chosen based on previous experience and standards in the field. For scRepli-seq analysis, we selected a sample size that ensures capturing cells at various stages throughout the S phase (Takahashi et al., Nature Genetics 2019). All sample sizes are shown in the figures or legends.
Data exclusions	For scRepli-seq analyses, we treated the data as described in Miura et al., Nature Protocols 2020. From a total of 120 single-cell data sets of wild-type NSCs, we excluded 15 that did not pass the 'MAD score' QC, 1 that showed problematic replication timing (RT) data distribution by the manhattan distance, as well as 6 with X chromosome abnormalities and used the remaining 98 data sets for downstream scRepli-seq analysis (4 G1-phase cells and 94 S-phase cells). From a total of 96 single-cell data sets of SmcHD1 mutant NSCs, we excluded 7 that did not pass the 'MAD score' QC and used the remaining 89 data sets for downstream scRepli-seq analysis (4 G1-phase cells and 85 S-phase cells).<br><br>For Xist RNA-FISH quantification, we excluded cells that were unclassified from the analysis (which were less than 1% of total cell analyzed). For a sequential Xist RNA-FISH and DNA-FISH experiment, we first excluded cells that did not show signal from Xist or show more than one Xist cloud/nucleus based on RNA-FISH results from the analyses. Next, we excluded cells that showed more than two spots from either SD, SI, or CL DNA-FISH signals near the Xi from the analyses.
Replication	All experiments, except for scRepli-seq and RT-PCR, were replicated at least twice, and in all cases, data sets exhibited high correlation among the replicates, assuring data reproducibility. Biological replicates represented by cells from different passages and different collected date.
Randomization	Randomization is not relevant to this study because no comparisons between experimental groups were made.
Blinding	Blinding was not relevant to this study because all metrics were derived from absolute quantitative methods without human subjectivity.

Reporting for specific materials, systems and methods

We require information from authors about some types of materials, experimental systems and methods used in many studies. Here, indicate whether each material, system or method listed is relevant to your study. If you are not sure if a list item applies to your research, read the appropriate section before selecting a response.

Materials & experimental systems

n/a	Involved in the study
☐	☒ Antibodies
☐	☒ Eukaryotic cell lines
☒	☐ Palaeontology and archaeology
☐	☒ Animals and other organisms
☒	☐ Clinical data
☒	☐ Dual use research of concern

Methods

n/a	Involved in the study
☒	☐ ChIP-seq
☐	☒ Flow cytometry
☒	☐ MRI-based neuroimaging

Antibodies

Antibodies used	Primary antibody:<br>1. Anti-BrdU antibody for BrdU-IP Repli-seq profiles (dilution to 12.5 ug/ml by PBS) from BD Biosciences Pharmingen (cat. 555627)<br>2. Anti-SmcHD1 antibody for western blot (1:500 dilution) from Sigma (cat. HPA039441; lot K106669)<br>3. Anti-alpha tubulin antibody for western blot (1:1000 dilution) from Abcam (cat. ab7291; lot GR138941-3)<br>4. Anti-Nestin antibody for immunostaining (1:200 dilution) from Wako (cat. 7A3)
Validation	All antibodies are commercially available and have associated datasheets from the supplier.<br>1. Anti-BrdU antibody was validated to immunoprecipitate BrdU-containing DNA and used for the previous study (Takahashi et al., Nature Genetics 2019)<br>2. Anti-SmcHD1 antibody was validated by western blot (https://www.sigmaaldrich.com/JP/ja/product/sigma/hpa039441)<br>3. Anti-alpha tubulin antibody was validated by western blot (https://www.abcam.com/products/primary-antibodies/alpha-tubulin-antibody-dm1a-loading-control-ab7291.html)<br>4. Anti-Nestin antibody was validated by immunostaining (https://labchem-wako.fujifilm.com/jp/product/detail/W01W0101-2684.html)

Eukaryotic cell lines

Policy information about cell lines and Sex and Gender in Research

Cell line source(s)	The JB4/EI7HZ2 mESC line (Matsuura et al., Front. Cell Dev. Biol. 2021) originated from Prof. Takashi Sado's laboratory at Kindai University, Japan. The human TERT-RPE1 cell line (Clontech, C4001-1) was obtained from Prof. Chikashi Obuse's laboratory at Osaka University, Japan.
Authentication	Karyotype (JB4/EI7HZ2) and SNPs (JB4) were verified using an NGS technology. SmcHD1 mutant JB4/EI7HZ2 mESC line generated in this study was validated by PCR, sequencing, and western blot. Differentiated wild-type and SmcHD1 mutant JB4/EI7HZ2 NSCs generated in this study were validated by RT-PCR, immunostaining, and RNA-seq.
Mycoplasma contamination	The cell lines were not tested for mycoplasma contamination.
Commonly misidentified lines<br>(See ICLAC register)	No commonly misidentified cell lines were used.

Animals and other research organisms

Policy information about studies involving animals; ARRIVE guidelines recommended for reporting animal research, and Sex and Gender in Research

Laboratory animals	EpiSCs and MEFs were isolated from E6.5 and E12.5 mouse embryos, respectively. EpiSCs were derived in the presence of IWP-2 according to Sugimoto et al., Stem Cell Reports 2015.<br>The housing conditions for the mice are as follows: they are exposed to a dark/light cycle with darkness from 19:00 to 7:00 and light from 7:00 to 19:00 (at an intensity of 200 lux). The temperature is maintained at 22±2 °C, and the humidity level is kept at 50±10%.
Wild animals	The study did not involve wild animals.
Reporting on sex	Female.
Field-collected samples	The study did not involve samples collected from the filed.
Ethics oversight	The animals were housed in environmentally controlled rooms, and all the experimental procedures using animals were approved by the Institutional Animal Care and Use Committee of Kindai University.

Note that full information on the approval of the study protocol must also be provided in the manuscript.

Flow Cytometry

Plots

Confirm that:

☒ The axis labels state the marker and fluorochrome used (e.g. CD4-FITC).

☒ The axis scales are clearly visible. Include numbers along axes only for bottom left plot of group (a 'group' is an analysis of identical markers).

☒ All plots are contour plots with outliers or pseudocolor plots.

☒ A numerical value for number of cells or percentage (with statistics) is provided.

Methodology

Sample preparation	Cells were fixed with 75% ethanol, and then stained with propidium iodide (PI) to assess the DNA content of the cells as previously described (Takahashi et al., Nature Genetics 2019). DNA content was analyzed using Sony SH800 cell sorter.
Instrument	Sony SH800 Cell sorter (SONY).
Software	The data was collected by Cell Sorter Software version 2.1.6.
Cell population abundance	1,000,000-2,000,000 cells were acquired for each sample. Around 90% of the cells showed the typical cell cycle profile.
Gating strategy	An FSC/BSC gate was used for gating the population of cells to exclude cell debris. Then, a PI gate was used to exclude doublet cells. The gates for sorting the G1 or desired S-phase fractions were defined on the PI histogram. For BrdU-IP, cells were sorted with the purity mode of the SH800 Cell sorter. For scRepli-seq, cells were sorted using the single-cell mode.

☒ Tick this box to confirm that a figure exemplifying the gating strategy is provided in the Supplementary Information.
